# Supplementary material for: Stimuli-Responsive Drug Delivery of Doxorubicin Using Magnetic Nanoparticle Conjugated Poly(ethylene glycol)-g-Chitosan Copolymer
Source: Int J Mol Sci. 2021 Dec 6;22(23):13169. doi: 10.3390/ijms222313169 (PMC8658650; doi:10.3390/ijms222313169)
Supplement: Supplementary file 1 [file ijms-22-13169-s001.zip › ijms-1468689-supplementary.pdf]

International Journal of
Molecular Sciences

MDPI

Supplementary materials

Experimental

Synthesis DOX-conjugated ChitoPEG magnetic nanoparticles

ChitoPEG copolymer: Synthesis of ChitoPEG copolymer was performed as reported previously [24]. Briefly, 180mg of WSC dissolved in 5 ml of deionized water was mixed with 10ml DMSO. To this solution, MePEG-NHS (500mg) was added and then magnetically stirred for 2 days. This solution was introduced into dialysis membrane (M.W. cut-off size, 8,000 g/mol) and then dialyzed against 1L deionized water for 3 days to remove unreacted MePEG and byproducts. To avoid saturation, water was exchanged every 3h intervals. Resulting solution lyophilized for 3 days and yellowish powder was obtained. Lyophilized solid was further purified by precipitation into excess chloroform and then dried under vacuum for 2 days. After that, this solid (ChitoPEG copolymer) was restored in 4°C until used for synthesis or analysis.

ChitoPEG-DOX conjugates: 21.5 mg of DOX HCl was dissolved in 5 ml of DMSO with one drop of TEA and then 15mg of CSSC-NHS was added (DOX.HCl/CSSC-NHS = 1/1 molar ratio). This solution was magnetically stirred for 6h to make DOX-CSSC-NHS conjugates. 193mg of ChitoPEG copolymer dissolved in 5ml deionized water was mixed with 10ml of DMSO and then this solution was added to DOX-CSSC-NHS solution. Mixed solution was magnetically stirred for 48h. After that, this solution was dialyzed against deionized water to remove unreacted DOX-CSSC-NHS and byproducts for 2 days with exchange of water at 3h intervals. Then, dialyzed solution was lyophilized for 3 days and ChitoPEG/DOX conjugates were observed.

Characterization of conjugates

^{1}H NMR spectra (500 mHz superconducting Fourier transform (FT)-NMR spectrometer, Varian Unity Inova 500 MHz NB High-Resolution FT NMR; Varian Inc., Santa Clara, CA) was used to monitor the synthesis of conjugates. To measure the ^{1}H NMR spectra, conjugates were dissolved in D_2O, DMSO or D_2O/DMSO mixtures.

Results and discussion

Figure S1 shows the synthesis scheme of ChitoPEG copolymer and ^{1}H NMR spectra. As shown in Figure s1, 3.2~3.8 ppm peak in ^{1}H -NMR-spectra is estimated to be attributable to the ethylene protons, the methoxyl group of MePEG, and H-2 to H-6 of chitosan, respectively(Figure s1). The observed peak near 2.5 ppm is assigned to originate from NH_2 in chitosan. In ^{1}H-NMR of Doxorubicin (Figure S2), characteristic peaks due to hydrogens 7, 8, and 9 in the benzene ring were observed at 7.5~8.0ppm. In dithiodipropionicacid–NHS ^{1}H-NMR, the peaks at 5.4-5.9 ppm for hydrogens 19 and 2.6-3.2 for hydrogens 20 were observed, respectively. In the analysis of ^{1}H-NMR spectra of DOX-CSSC-NHS, a sharp peak due to the 10-methoxy group of doxorubicin was observed at 3.4 ppm, and a peak due to hydrogens 11-15 was observed between 3.0 and 4.5 ppm. Based on ^{1}H NMR spectra, degree of substitution value was approximately 10.8 glucose unit/1PEG molecule. DOX HCl was reacted with dithiodipropionic acid NHS ester (Figure S2).

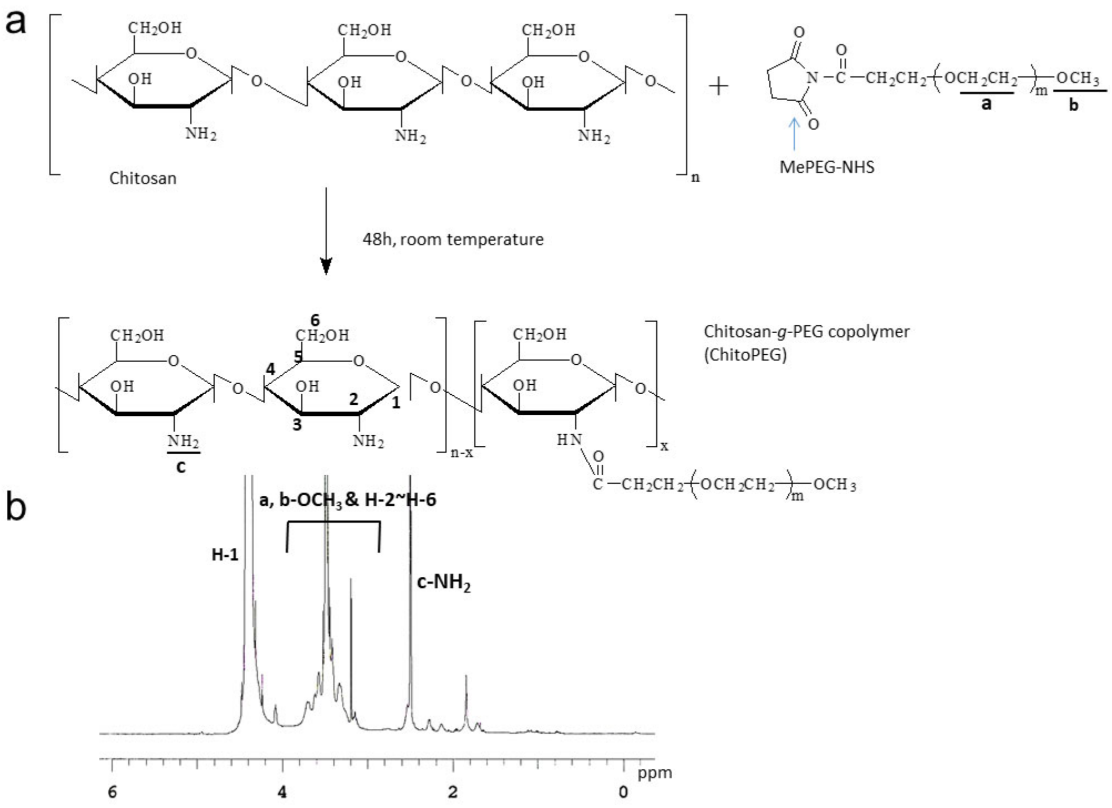

Figure S1. (**a**) Synthesis scheme and (**b**) ^{1}H NMR spectra of ChitoPEG graft copolymer.

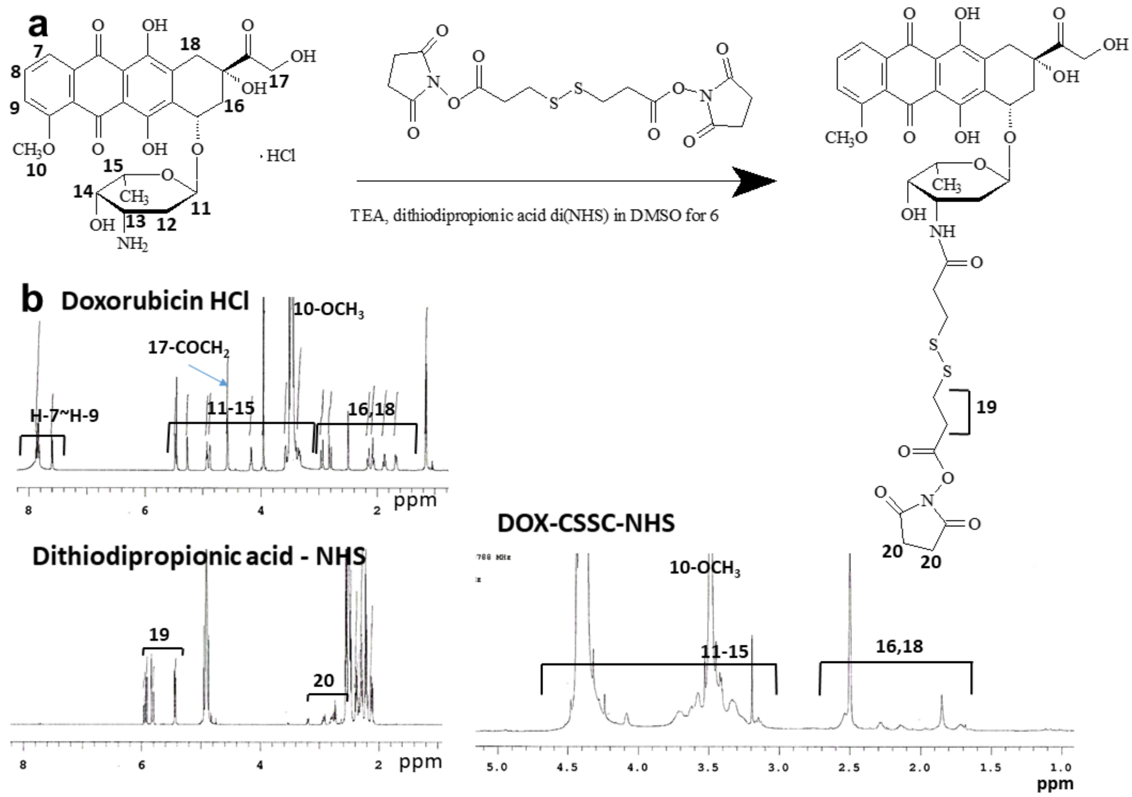

Figure S2. (**a**) Synthesis scheme and (**b**) ^{1}H NMR spectra of DOX-dithiodipropinic acid N-hydroxysucinimide ester.
